# Gene Expression Profiles Identify Inflammatory Signatures in Dendritic Cells

**DOI:** 10.1371/journal.pone.0009404

**Published:** 2010-02-24

**Authors:** Anna Torri, Ottavio Beretta, Anna Ranghetti, Francesca Granucci, Paola Ricciardi-Castagnoli, Maria Foti

**Affiliations:** 1 Department of Biotechnology and Bioscience, University of Milano-Bicocca, Milan, Italy; 2 Singapore Immunology Network, Singapore, Singapore; Fundação Oswaldo Cruz, Brazil

## Abstract

Dendritic cells (DCs) constitute a heterogeneous group of antigen-presenting leukocytes important in activation of both innate and adaptive immunity. We studied the gene expression patterns of DCs incubated with reagents inducing their activation or inhibition. Total RNA was isolated from DCs and gene expression profiling was performed with oligonucleotide microarrays. Using a supervised learning algorithm based on Random Forest, we generated a molecular signature of inflammation from a training set of 77 samples. We then validated this molecular signature in a testing set of 38 samples. Supervised analysis identified a set of 44 genes that distinguished very accurately between inflammatory and non inflammatory samples. The diagnostic performance of the signature genes was assessed against an independent set of samples, by qRT-PCR. Our findings suggest that the gene expression signature of DCs can provide a molecular classification for use in the selection of anti-inflammatory or adjuvant molecules with specific effects on DC activity.

## Introduction

Dendritic cells (DCs) are bone marrow-derived cells present in all lymphoid and non lymphoid organs. They play a role in immune regulation, inducing tolerance and preventing autoimmunity, inducing anti-tumor immunity and protecting against infectious agents. DCs constitute a heterogeneous group of cells with different origins (both myeloid and lymphoid), anatomic locations, cell surface phenotypes and functions. Within the different DCs subtypes, myeloid DCs are also the most efficient antigen-presenting cells in the induction of naive, memory, effector and regulatory T-cell responses [1]–[3].

DCs have several pattern recognition receptors (such as Toll-like receptors). During infection or inflammation, these receptors interact with microbe-associated molecules (such as LPS, bacterial DNA and double-stranded RNA), resulting in DC activation [4], [5]. Endogenous TLR ligands are also released in conditions of inflammation, such as cell injury, and induce similar activation programs [6], [7]. These programs affect various DC functions, such as migration to draining lymph nodes for antigen presentation, costimulation and the production of a specific cytokine profile determining the type of T-cell response to be developed. This process is known as maturation and it enables DCs to initiate and direct the acquired immune system (B and T cells) and, ultimately, to mount an antigen (Ag)-specific response [8].

Global transcriptomic analysis has recently been shown to be a powerful approach yielding new insight into the biology of specific cell subsets or tissues, by providing information about their specific gene expression programs [9]–[12]. Moreover, the analysis of genome-wide expression profiles is now a widely used technique for the identification of diagnostic markers of various disease states, outcomes, or responses to treatment [13]–[17]. Markers are selected by scoring each individual gene on the basis of the extent to which its expression pattern discriminates between different classes of disease or between cases and controls. The disease status of new patients is predicted with classifiers tuned to the expression levels of the marker genes. One potential problem with expression-based classification is that cellular heterogeneity within tissues and genetic heterogeneity between samples may decrease the discriminatory power of individual genes in complex diseases [18], [19]. As DCs are involved in various diseases involving the immune system, from inflammatory diseases to cancer, the identification of molecular markers in DCs specific to inflammation is of potential clinical and pharmaceutical value.

A number of time course and end points studies of the DCs activation process have been published to describe the dynamic process of interaction among gene transcripts that are important for controlling many of the observed changes that occur during the process of activation/maturation [20]–[28]; however, these studies utilize analysis methods for differential gene expression and do not take into account class prediction methods. We applied a classification algorithm to derive a list of genes able to predict the DCs activation state. In this study, we identified a genetic signature of inflammation in mouse DCs. We chose to study mice, because they are widely used in models of many immunological diseases. These findings may lead to the identification of a prospective signature of inflammation and should increase our understanding of the biological processes underlying chronic inflammatory diseases.

## Results

### Sample Selection and Processing

In total, 115 samples were analyzed to develop a prognostic molecular assay of DC activation. Seventy-seven arrays were used for the training set and 38 were used for the testing set. We analyzed different samples of the DC line D1 [29] treated with inflammatory stimuli including bacteria (*Listeria monocytogenes*), helminths (*Schistosoma* eggs), protozoa (*Leishmania* promastigotes) and TLR ligands (LPS, poly I:C and zymosan) and samples of D1 cells treated with dexamethasone, *Schistosoma* SLA and *Leishmania* amastigotes, all of which are known to downregulate the inflammatory response [30], [31]. The microarray data used were either generated in this study or derived from previous experiments [23], [30], [32]. Table 1 describes the sample dataset used in this study. We amplified total RNA and hybridized it to an Affymetrix mouse MG-U74Av2 GeneChip oligonucleotide microarray containing 12,488 probe sets. The resulting microarray signal intensities for all 12,488 probe sets were normalized and the background was subtracted.

**Table 1:** Characteristic of the Microarray Data Set used.

Cell Type	Stimulus	N° of Arrays	Class Ass
DC D1	None	14	Non Inflamm
DC D1	DEX	6	Non Inflamm
DC D1	Leishmania Ama	8	Non Inflamm
DC D1	Shistosoma SLA	8	Non Inflamm
DC D1	CpG	10	Inflamm
DC D1	Leishmania Pro	8	Inflamm
DC D1	Listeria EGD	20	Inflamm
DC D1	LPS	8	Inflamm
DC D1	PAM3Cys	10	Inflamm
DC D1	Poly I:C	10	Inflamm
DC D1	Shistosoma Eggs	8	Inflamm
DC D1	Zymosan	15	Inflamm
Total		115	

### Multivariate Analysis Reveals the Existence of an Inflammatory State for Dendritic Cells

**Figure 1:**
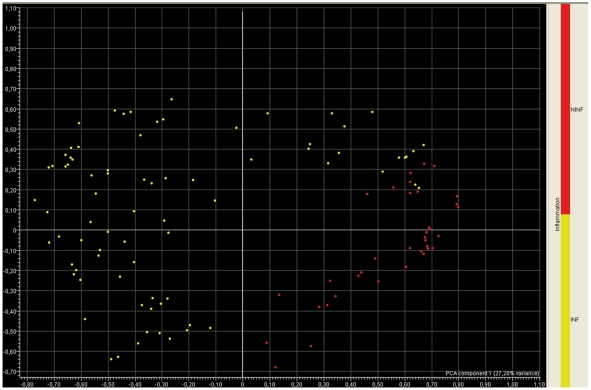
PCA score plot. Seventy-nine “inflammatory” observations and 36 “non inflammatory” observations (listed in Table 1) used to generate and test the random forest model. Genome-wide gene expression data were collected with DNA-microarray technology and the normalized hybridization signals were analyzed by PCA. A score plot with the first and second principal component axes is shown. Inflamed samples are mostly projected toward negative values of the first PC axis, whereas samples from controls and non inflamed samples are projected toward positive values. INF: Inflamed samples; NINF: non-inflamed samples.

Principal component analysis (PCA) makes it possible to visualize correlations in datasets by compressing information into a small number of dimensions. PCA was carried out on the data for DCs treated with stimuli inducing activation via various receptors, including the Toll-like receptors. Projection of the samples onto a plane corresponding to the first two dimensions derived from PCA resulted in a clear separation along the first dimension (Figure 1). Control and experimental samples treated with anti-inflammatory stimuli were projected toward positive values of the first dimension and samples with signs of activation were projected toward negative values. The PCA data suggest that DCs in different functional states could be separated on the bases of differences in inflammatory stimulation and that the inflamed samples could be isolated from all the other stimuli in a typical two-classes partitioning.

### Data Analysis Strategy for the Selection of Classifier Genes

We carried out a step-wise analysis to determine whether it was possible to select a gene expression signature of inflammation: (a) an expression index was calculated with RMA [33]; (b) sample classification: genes capable of discriminating between the two groups were identified by comparing groups of samples in the inflammatory group with those in the non inflammatory group (training set); (c) independent validation of classifier genes: the genes selected were used to classify an independent group of samples (testing set); (d) validation of the genetic signature by quantitative RT-PCR (qRT-PCR) on independent DCs samples prepared with different stimuli. The procedure used for the selection and preparation of microarrays is shown in Figure S1.

### Transcriptional Signatures Discriminate between Inflammatory and Steady State Cellular Phenotypes

**Figure 2:**
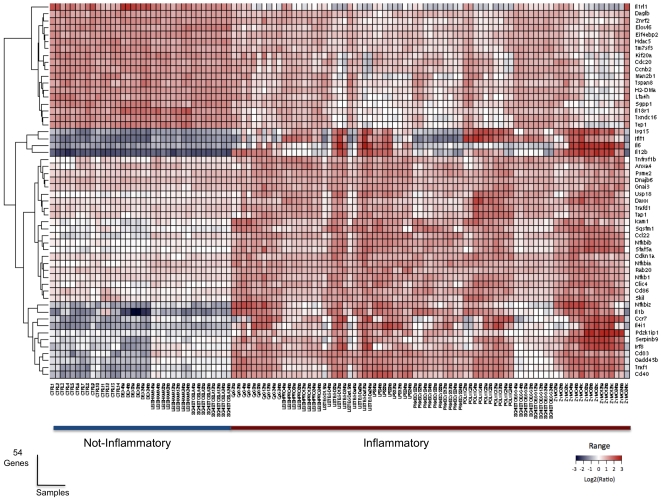
Heat map of the dendritic cell-specific signature. Genes with different levels of expression in DCs treated with inflammatory stimuli and in those treated with anti-inflammatory molecules. Each column represents a sample and each row represents a gene. Levels of gene expression are indicated on a color scale, with red corresponding to the highest level of expression and blue corresponding to the lowest level. The Log2 ratio is expressed with respect to the mean expression level of each gene.

Raw intensity values from microarray hybridization were normalized with the robust multiarray average in the R-package for statistical computing (available at www.R-project.org). A random forest classification model was built from a training set (50 observations in conditions of inflammation, 27 observations in non inflammatory conditions) obtained from the genome-wide gene expression analysis of DCs incubated with different stimuli. All the samples were assigned to training or testing sets: two thirds of the samples (n = 77) were assigned to the training sets, the remaining third being assigned to testing sets (n = 38; Figure S1). The results obtained for the untreated samples and those treated with non inflammatory stimuli were very similar and these two groups of samples were therefore considered to belong to the same class (data not shown). This approach resulted in the identification of 54 genes distinguishing accurately between the two classes of samples, as demonstrated by analysis with the testing set (data not shown; Table 2). The molecular signature was illustrated with a heat map based on Euclidean distance (Figure 2). We identified 18 genes downregulated by inflammation and 36 upregulated by inflammation. Unsupervised clustering analysis confirmed the robustness of the set of genes identified, with very clear distinction between samples treated with and without inflammatory stimuli (Figure 2). Gene Ontology was used to classify the modulated genes in terms of function. The genes selected encoded proteins involved mainly in the immune system process (44%), cell differentiation (44%), cell death (30%), and regulation of biological process (55%), as shown in Table 3.

**Table 2:** Fifty-four Genes able to predict Inflammatory Signatures in DC.

Affymetrix ID	Gene Title	Gene Symbol
100423_f_at	SIGNAL TRANSDUCER AND ACTIVATOR OF TRANSCRIPTION 5A	STAT5A
100540_at	LEUKOTRIENE A4 HYDROLASE	LTA4H
100584_at	ANNEXIN A4	ANXA4
100588_at	PROTEASOME (PROSOME, MACROPAIN) 28 SUBUNIT, BETA	PSME2
100779_at	INTERLEUKIN 12B	IL12B
100981_at	INTERFERON-INDUCED PROTEIN WITH TETRATRICOPEPTIDE REPEATS 1	IFIT1
101072_at	EUKARYOTIC TRANSLATION INITIATION FACTOR 4E BINDING PROTEIN 2	EIF4EBP2
101144_at	INTERLEUKIN 18 RECEPTOR 1	IL18R1
101995_at	SEQUESTOSOME 1	SQSTM1
102218_at	INTERLEUKIN 6	IL6
102310_at	CHEMOKINE (C-C MOTIF) LIGAND 22	CCL22
102779_at	GROWTH ARREST AND DNA-DAMAGE-INDUCIBLE 45 BETA	GADD45B
102831_s_at	CD86 ANTIGEN	CD86
103035_at	TRANSPORTER 1, ATP-BINDING CASSETTE, SUB-FAMILY B (MDR/TAP)	TAP1
103040_at	CD83 ANTIGEN	CD83
103254_at	TRAF TYPE ZINC FINGER DOMAIN CONTAINING 1	TRAFD1
103402_at	TRANSMEMBRANE 7 SUPERFAMILY MEMBER 3	TM7SF3
103486_at	INTERLEUKIN 1 BETA	IL1B
103494_at	TETRASPANIN 8	TSPAN8
103665_at	ELOVL FAMILY MEMBER 6, ELONGATION OF LONG CHAIN FATTY ACIDS (YEAST)	ELOVL6
104149_at	NUCLEAR FACTOR OF KAPPA LIGHT CHAIN GENE ENHANCER IN B-CELLS INHIBITOR, ALPHA	NFKBIA
104376_at	HISTONE DEACETYLASE 5	HDAC5
104418_at	ZINC AND RING FINGER 2	ZNRF2
104443_at	CHEMOKINE (C-C MOTIF) RECEPTOR 7	CCR7
160501_at	KINESIN FAMILY MEMBER 20A	KIF20A
160608_at	RAB20, MEMBER RAS ONCOGENE FAMILY	RAB20
161005_at	RIKEN CDNA 5730420B22 GENE	TXNDC16
92962_at	CD40 ANTIGEN	CD40
93092_at	HISTOCOMPATIBILITY 2, CLASS II, LOCUS DMA	H2-DMA
93367_at	TELOMERASE ASSOCIATED PROTEIN 1	TEP1
93448_at	RIKEN CDNA E330036I19 GENE	DAGLB
94186_at	TNF RECEPTOR-ASSOCIATED FACTOR 1	TRAF1
94256_at	CHLORIDE INTRACELLULAR CHANNEL 4 (MITOCHONDRIAL)	CLIC4
94294_at	CYCLIN B2	CCNB2
94501_at	SPHINGOSINE-1-PHOSPHATE PHOSPHATASE 1	SGPP1
94752_s_at	SKI-LIKE	SKIL
94814_at	GUANINE NUCLEOTIDE BINDING PROTEIN, ALPHA INHIBITING 3	GNAI3
94881_at	CYCLIN-DEPENDENT KINASE INHIBITOR 1A (P21)	CDKN1A
94928_at	TUMOR NECROSIS FACTOR RECEPTOR SUPERFAMILY, MEMBER 1B	TNFRSF1B
95024_at	UBIQUITIN SPECIFIC PEPTIDASE 18	USP18
96120_at	DNAJ (HSP40) HOMOLOG, SUBFAMILY B, MEMBER 6	DNAJB6
96125_at	FAS DEATH DOMAIN-ASSOCIATED PROTEIN	DAXX
96319_at	RIKEN CDNA 2310042N09 GENE	CDC20
96515_at	INTERLEUKIN 4 INDUCED 1	IL4I1
96752_at	INTERCELLULAR ADHESION MOLECULE	ICAM1
96935_at	PDZK1 INTERACTING PROTEIN 1	PDZK1IP1
98002_at	INTERFERON REGULATORY FACTOR 8	IRF8
98405_at	SERINE (OR CYSTEINE) PEPTIDASE INHIBITOR, CLADE B, MEMBER 9	SERPINB9
98427_s_at	NUCLEAR FACTOR OF KAPPA LIGHT CHAIN GENE ENHANCER IN B-CELLS 1, P105	NFKB1
98500_at	INTERLEUKIN 1 RECEPTOR-LIKE 1	IL1RL1
98822_at	INTERFERON, ALPHA-INDUCIBLE PROTEIN	ISG15
98988_at	NUCLEAR FACTOR OF KAPPA LIGHT POLYPEPTIDE GENE ENHANCER IN B-CELLS INHIBITOR, ZETA	NFKBIZ
99562_at	MANNOSIDASE 2, ALPHA B1	MAN2B1
99982_at	NUCLEAR FACTOR OF KAPPA LIGHT CHAIN GENE ENHANCER IN B-CELLS INHIBITOR, BETA	NFKBIB

**Table 3:** Upregulated genes are grouped functionally based on ontologies, and levels of significance are shown.

GO Biological Process	N. of Genes	%	pValue	Gene symbol	Benjamini	FDR
**GO:0002376-immune system process**	16	44,44	2,69E-11	*Psme2, Nfkbia, Stat5a, IL1b, Tap1, IL12b, Irf8, Ccl22, Sqstm1, Il6, Nfkb1, CD40, Cdkn1a, Ifit1, Ccr7, Isg15*	1,39E-07	5,13E-08
**GO:0030154- cell differentiation**	16	44,44	9,5E-07	Nfkbia, *Skil*, *Traf1*, Stat5a, *Clic4*, Gadd45b, Il12b, Irf8, *Tnfrsf1b*, *Serpinb9*, Sqstm1, Il6, Nfkb1, Cd40, Cdkn1a, *Daxx*	1,23E-03	1,82E-03
**GO:0008219- cell death**	11	30,56	1,16E-06	Traf1, Stat5a, Sqstm1, Il6, Nfkb1, Cd40, Cdkn1a, *Gadd45b*, Tnfrsf1b, Serpinb9, Daxx	1,21E-03	2,23E-03
**GO:0001816-cytokine production**	6	16,67	3,86E-06	Stat5a, Il1b, Il6, Nfkb1, Cd40, Il12b	2,86E-03	7,37E-03
**GO:0012501- programmed cell death**	10	27,78	7,97E-06	Traf1, Stat5a, Sqstm1, Il6, Nfkb1, Cd40, Cdkn1a, Gadd45b, Serpinb9, Daxx	4,59E-03	1,52E-02
**GO:0006954- inflammatory response**	6	16,67	9,43E-05	Ccl22, Stat5a, Il1b, Il6, *Nfkbiz*, Tnfrsf1b	3,01E-02	1,80E-01
**GO:0032502- developmental process**	17	47,22	9,91E-05	Nfkbia, Skil, Traf1, Stat5a, Clic4, *Anxa4*, Gadd45b, Il12b, Irf8, Tnfrsf1b, Serpinb9, Sqstm1, Il6, Nfkb1, Cd40, Cdkn1a, Daxx,	2,98E-02	1,89E-01
**GO:0050789- regulation of biological process**	20	55,56	1,13E-04	Nfkbia, Traf1, Stat5a, Il1b, Clic4, Nfkbiz, *Icam1*, Gadd45b, Il12b, Irf8, Tnfrsf1b, Serpinb9, *Dnajb6*, Sqstm1, Il6, Nfkb1, Cd40, Cdkn1a, Daxx, *Gnai3*	3,21E-02	2,16E-01
**GO:0050896- response to stimulus**	17	47,22	4,73E-04	Nfkbia, Stat5a, Il1b, Nfkbiz, Tap1, Il12b, Irf8, Tnfrsf1b, Ccl22, Sqstm1, Il6, *Cd86*, Cd40, Cdkn1a, Ifit1, Ccr7, Isg15	7,87E-02	9,01E-01
**GO:0032020- ISG15 protein conjugation**	2	5,56	1,03E-02	*Usp18*, Isg15	5,69E-01	1,80E+01
**GO:0007154-cell communication**	16	44,44	1,49E-02	Nfkbia, Traf1, Stat5a, Il1b, *Rab20*, Gadd45b, Il12b, Tnfrsf1b, Ccl22, Sqstm1, Il6, Nfkb1, Cd40, Ccr7, Daxx, Gnai3	6,86E-01	2,49E+01

The downregulated genes were found to encode proteins involved in the biological pathways of cell division (Ccnb2, Kif20a, Cdc20), lipid metabolic process (Daglb, Elovl6, Lta4h, Sgpp1), defense response (hdac5, Il1rl1, Il18r1, Lta4h), and metabolic processes (Txndc16, Hdac5, Il1rl1, Daglb, Lta4h, Tep1, Sgpp1, H2-DMa, Eif4ebp2, Man2b1, Elovl6, Znrf2, Cdc20). Two of these genes, Tm7sf3 and Tspan8, could not be functionally classified (Table 4). The upregulated genes encoded proteins involved in the immune system response (Psme2, Cd40, Ccl22, Il1b, Sqstm1, Tap1, Il6, Il12b, Ifit1, Ccr7, Irf8, Isg15, Nfkbia, Nfkb1, Stat5a, Cdkn1a), cell death (Traf1, Stat5a, Sqstm1, Il6, Nfkb1, Cd40, Cdkn1a, Gadd45b, Tnfrsf1b, Serpinb9, Daxx), regulation of biological processes (Nfkbia, Traf1, Stat5a, Il1b, Clic4, Nfkbiz, Icam1, Gadd45b, Il12b, Irf8, Tnfrsf1b, Serpinb9, Dnajb6, Sqstm1, Il6, Nfkb1, Cd40, Cdkn1a, Daxx, Gnai3,) and cell differentiation (Nfkbia, Skil, Traf1, Stat5a, Clic4, Gadd45b, Il12b, Irf8, Tnfrsf1b, Serpinb9, Sqstm1, Il6, Nfkb1, Cd40, Cdkn1a, Daxx).

**Table 4:** Downregulated genes are grouped functionally based on ontologies.

GO Biological Process	N. of Genes	%	pValue	Gene symbol	Benjamini	FDR
**GO:0051301- cell division**	3	16,67	2,45E-02	*Ccnb2*, *Kif20a*, Cdc20	1	37,754
**GO:0006629- lipid metabolic process**	4	22,22	2,57E-02	Daglb, Elovl6, Lta4h, Sgpp1	1	39,157
**GO:defense response**	4	22,22	3,36E-02	Hdac5, Il1rl1, Il18r1, Lta4h	1	47,956
**GO:0008152- metabolic process**	13	72,22	3,38E-02	*Txndc16*, *Hdac5, Il1rl1*, *Daglb6, Lta4h, Tep1, Sgpp1, H2-DMa, Eif4ebp2, Man2b1, Elovl6*, *Znrf2, Cdc20*	1	48,194
**GO:0002376- immune system process**	4	22,22	5,10E-02	Hdac5, H2-DMa, Il1rl1, *Il18r1*	1	63,237
**GO:0045087- innate immune response**	2	11,11	9,56E-02	Il1rl1, Il18r1	1	85,352

### Real-Time Reverse Transcriptase (RT)-PCR Validation of Microarray Observations

**Figure 3:**
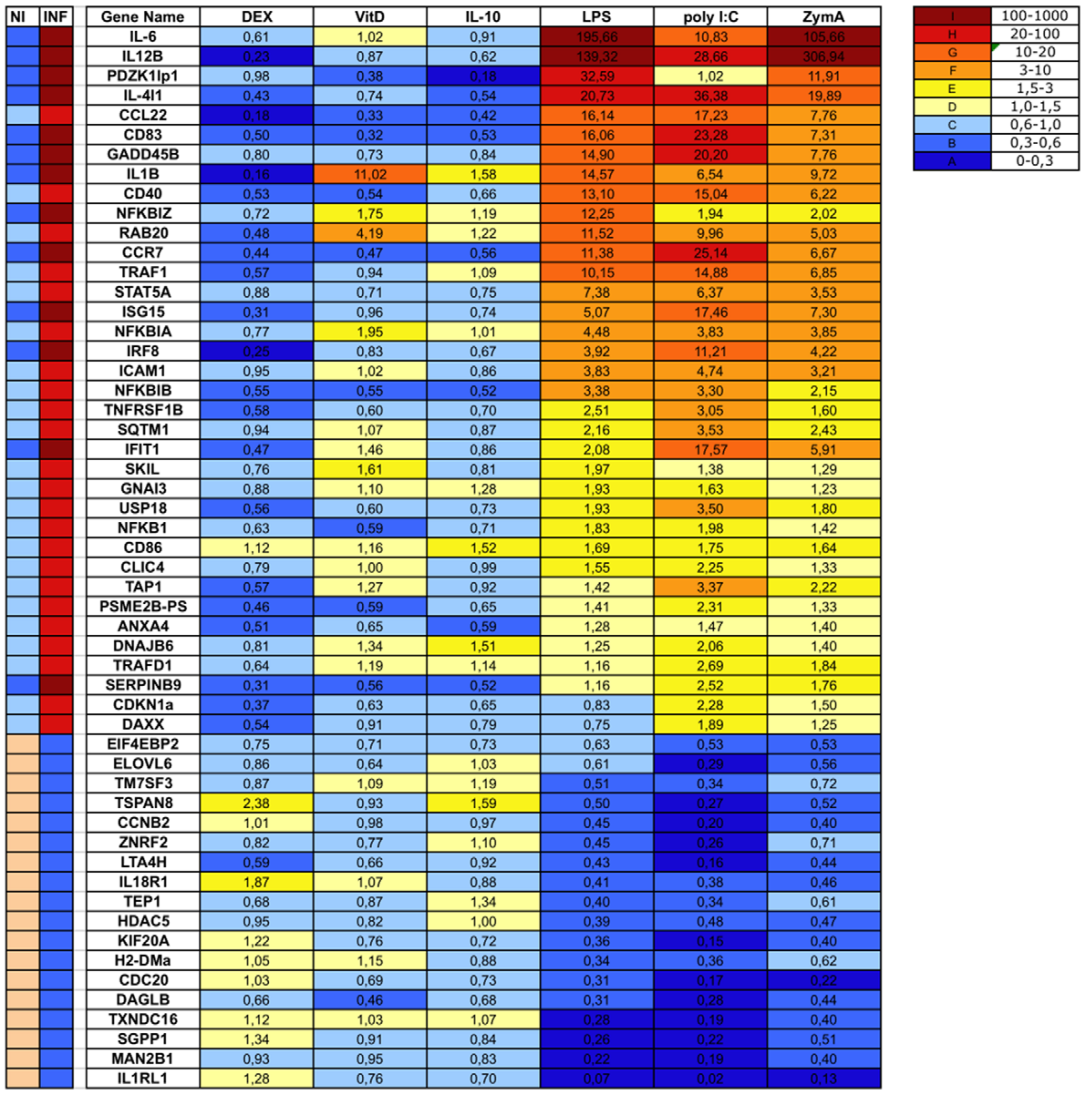
Sample validation by quantitative real-time PCR. qRT-PCR confirmation of classifier transcript levels in inflamed samples derived from DCs treated with TLR ligands (LPS, PolyI:C and zymosan) and in non inflamed samples treated with anti-inflammatory stimuli (dexamethasone, vitamin D and IL-10) for 24 h. Reactions were performed in two wells, normalized to 18s rRNA levels. The results in the table are expressed relative to the corresponding level of expression of each transcript in the untreated sample. Data are presented as mean fold changes in classifier gene transcript levels in three independent experiments per group. The columns in the left reflect the pattern of expression as determined by microarray analysis. NI: Non inflamed samples; INF; Inflamed samples.

Despite the accuracy of the classifier for random forest-based class assignment in a test set of DC samples, the mean expression index of transcripts was low. We therefore performed qRT-PCR to confirm the relative expression levels recorded for the DC samples with Affymetrix technology. We used the 18s rRNA gene as a housekeeping gene for the normalization of target gene expression. We prepared independent DC line D1 samples by treating the cells for 24 h with known signals, such as 10 µg/ml LPS, 20 µg/ml Poly I:C and 500 ng/ml zymosan, and with 10^−8^ M dexamethasone, 10^−8^ M vitamin D and 50 ng/ml IL10, which are known to have anti-inflammatory activity [34]–[38]. All the RNA samples in the study were converted to cDNA using the same reverse-transcription cocktail and procedure. The pattern of gene expression observed on qRT-PCR confirmed the microarray data. We assessed the predictive value of genes by calculating the median levels of expression for that gene in the known inflammatory and anti-inflammatory samples and then determining the mean expression level for the gene between the two classes. These threshold values were used to determine whether, for a given stimulus, the level of expression of the gene could be used to assign the sample to the correct class. We therefore subjected all the genes to the same test, under different stimulation conditions. A score of 1 was assigned if the expression level exceeded the mean expression value for an upregulated gene for a sample to be inflammatory or was less than the mean value for a sample to be anti-inflammatory. For repressed genes, we applied the opposite, a score of 1 was assigned if the expression level was less than the mean expression value for a sample to be inflammatory or was more than the mean value for a sample to be anti-inflammatory. A score of 0 was awarded in all other cases (Figure S2). Poly I:C stimulation was correctly classified by 94% (51/54) of the genes, whereas LPS and zymosan stimulations were correctly classified by 89% (48/54) and 85% (46/54) of the genes, respectively. As predicted, dexamethasone was the best anti-inflammatory reagent, correctly classified by 100% (54/54) of the genes. IL10 and vitamin D were identified as anti-inflammatory stimuli by 96% (52/54) and 89% (48/54) of the genes, respectively (Figure 3).

Vitamin D upregulated Il1b, Rab20, Nfkbia, Nfkbiz and Skil. These genes are upregulated by TLRs ligands (Figure 3). The effect of vitamin D on IL-1b gene expression has already been demonstrated in primary mouse keratinocytes and other cell types, whereas the induction of proteins involved in intracellular trafficking has not previously been shown [39], [40]–[42]. Rab20 was recently identified as a potential regulator of connexin 43 trafficking [43].

**Figure 4:**
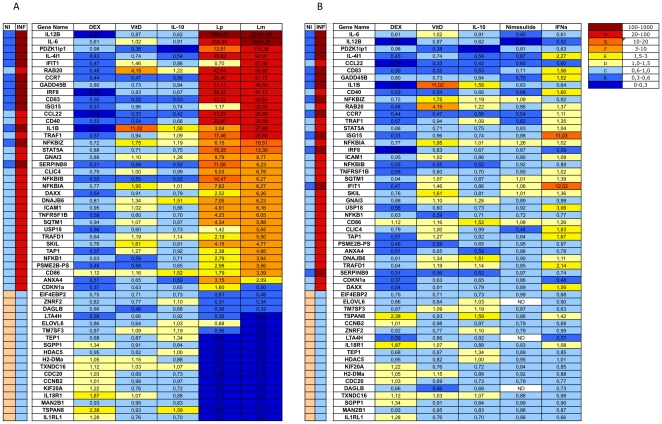
q-Real-time PCR analysis of gene expression on independent samples for class prediction. A) Expression levels of 54 genes in DCs treated with the bacteria *Listeria monocytogenes* (Lm) and *Lactobacillus paracasei* (Lp) for 24 h. B) DC samples treated with nimesulide and IFNα for 24 h. Comparison with cells treated with dexamethasone (DEX), IL-10 and vitamin D (VitD). Data are presented as mean fold changes in classifier gene transcript levels in three independent experiments per group. NI: Non inflamed samples; INF; Inflamed samples.

We then assessed the power of the selected genetic signatures to classify samples treated with different reagents probing different types of receptors. We treated DCs with live bacteria, such as *Listeria monocytogenes* and *Lactobacillus paracasei*, 10 µM nimesulide and 1000 U/ml IFNα to modify their functional state. As expected, live bacteria induced the strongest inflammatory signature response in DCs (Figure 4A): *Listeria monocytogenes* and *Lactobacillus paracasei* were correctly classified as inflammatory by 98% (53/54) and 94% (51/54) of the genes, respectively. *Listeria monocytogenes* is known to induce the production of type I IFNs. We therefore expected this bacterium to induce the interferon-responsive genes Ifit1 and Isg15. Levels of transcription for Il1b, Pdzk1ip1, Isg15, Ifit1 and Usp18 were strongly reduced by *Lactobacillus paracasei*. We therefore conclude that *Lactobacillus paracasei* is less able to induce a type I IFN response than other genes of the inflammatory signature, suggesting that commensal bacteria may specifically modulate inflammatory genes in DCs (Figure 4A). Finally, we measured the effect of IFNα and nimesulide treatment on DCs. We compared the results obtained with nimesulide and IFNα with the signatures obtained with dexamethasone, IL10 and vitamin D (Figure 4B). Nimesulide and IFNα were classified as anti-inflammatory by 98% (53/54) and 80% (43/54) of the genes, respectively. Isg15, Ifit1, Usp18 are known to be regulated by IFNα, whereas Clic4, Trafd1 and Il4i1 have not been shown to be affected by IFNα. This confirms the role of IFNα in the regulation of DC activity [44], [45].

We were able to classify samples treated with IFNα, Il10, vitamin D as non inflammatory with this system, whereas live bacteria (*Listeria* and *Lactobacillus*) were classified as inflammatory on the basis of the strong inflammatory signatures induced. LPS and Poly I:C were found to be stronger inducers of DC activation than zymosan (46/54), at least in our experimental conditions. Zymosan has also been shown to induce a regulatory phenotype in DCs [46].

### Transcription Signatures Are Dendritic Cell-Specific

**Figure 5:**
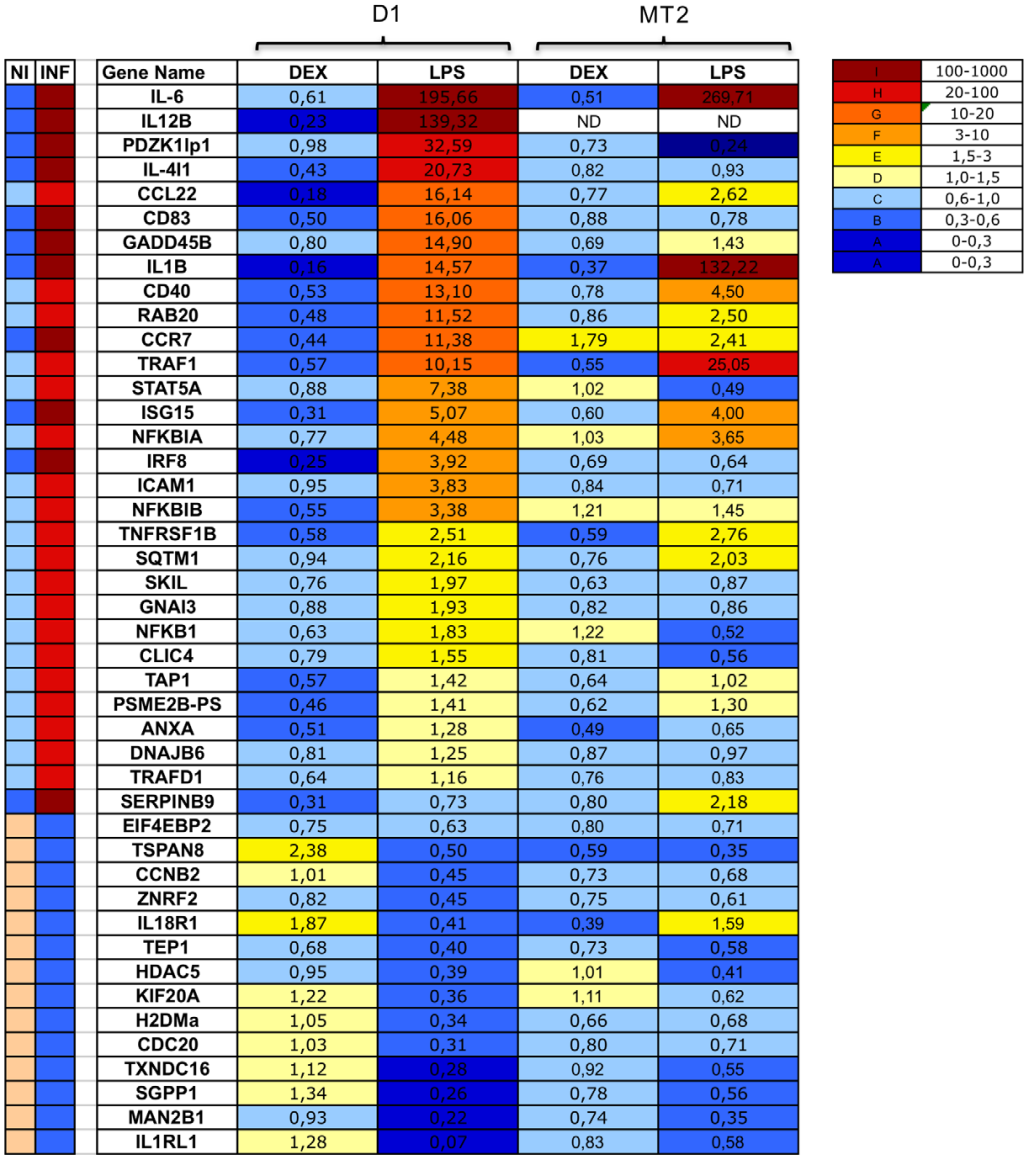
Specificity of the genetic signature. Real-time PCR confirmation of 44 inflammatory signature genes in the DC line D1 and the absence of this signature in MT2 cells. Both cell lines were treated with LPS (10 µg/ml) and 10^−8^ M dexamethasone (DEX) for 24 h. Data are presented as mean fold changes in classifier gene transcript levels in three independent experiments per group.

We assessed the specificity of the classifier genes by analyzing 44 of the 54 genes that gave consistent results for different samples by qRT-PCR, in the macrophage cell line MT2 [47]. The MT2 cells were stimulated with 10^−8^ M dexamethasone or 10 µg/ml LPS. The genes of the inflammation signature were not well expressed in MT2 cells if compared to the D1 cells (Figure 5). Only 41% (18/44) of the genes showed the expected pattern of change in expression in response to LPS stimulation in MT2 cells. Indeed, some genes displayed opposite patterns of expression in the two cell lines tested. Il18r1 was induced in MT2 cells but downregulated in DCs in response to LPS. We therefore conclude that the inflammatory signature identified in this study is specific to DCs.

### Classifier Genes Can Be Used to Predict the Inflammatory Process in DCs *In Vivo*

Preclinical animal models of inflammation and infections have become important tools for improving our understanding of the regulation of inflammatory reactions in general and for the development of novel treatment strategies to modulate excessive, deleterious inflammatory reactions. We measured the gene expression signature associated with inflammation in *ex vivo* splenic DCs derived from mice treated with the endotoxin LPS, and dexamethasone with the aim of converting our *in vitro* DC assay into a useful tool for preclinical mouse models of inflammatory diseases.

**Figure 6:**
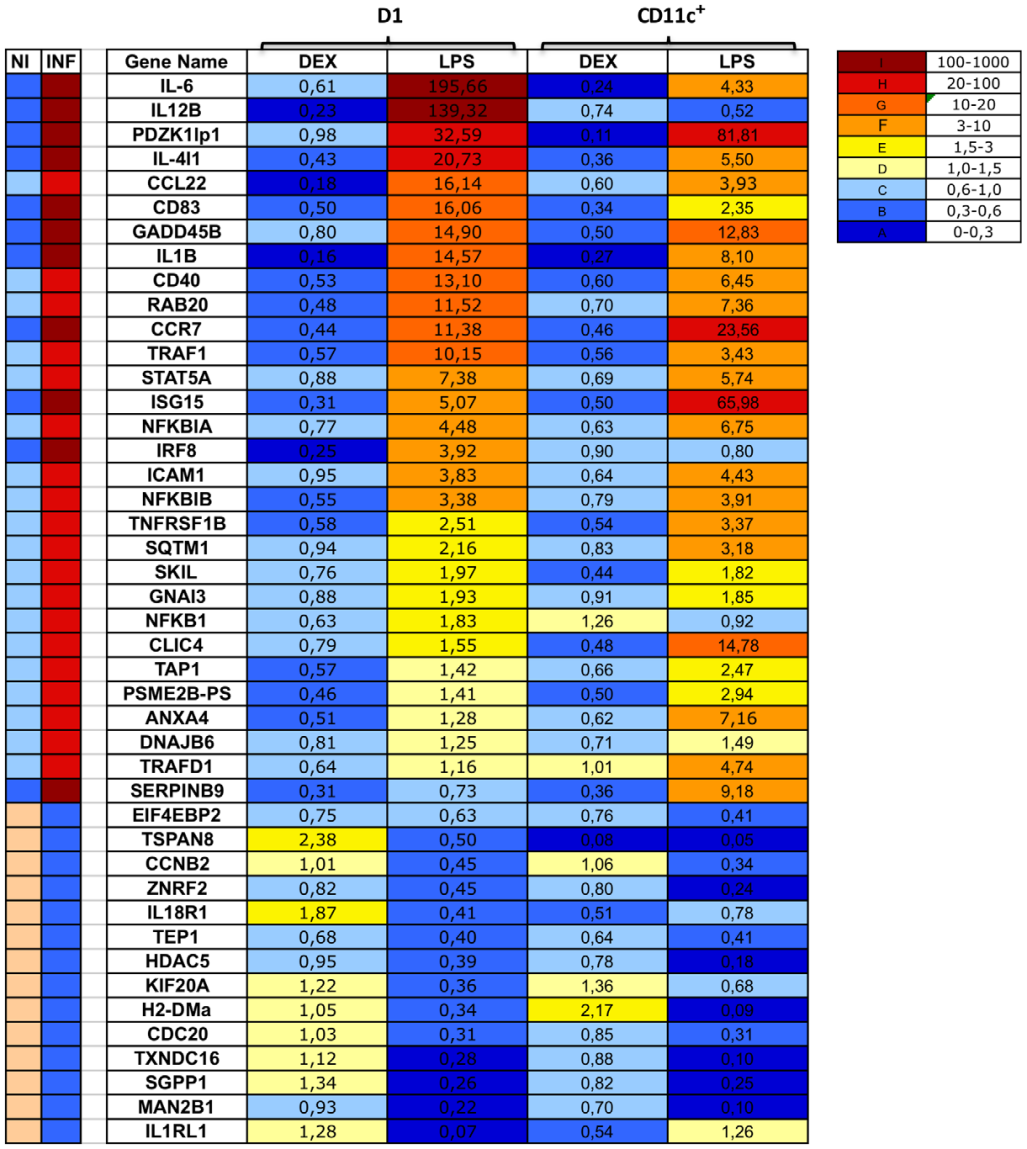
*In vivo* validation of the DC-specific inflammatory signature. C57BL/6 mice were treated with LPS and, after 5 h, CD11c+ cells were isolated and tested for the inflammatory signature. Data are presented as mean fold changes in classifier gene transcript levels in three independent experiments.

Splenic DCs detect antigens derived from the blood and are widely used as a model system for testing treatments that affect DC recruitment or for detecting DC activation during systemic infection. We analyzed the pattern of expression of the 44 genes in splenic DCs *in vivo*. We treated a group of mice with 50 µg of LPS/mouse or dexamethasone and, after 5 h, CD11c^+^ cells were purified from spleen by magnetic bead separation. Cell purity was checked by FACS analysis and 90% of the cells were found to be CD11c^+^ (data not shown) then assessed the prognostic value of the 44 genes by qRT-PCR: 91% (40/44) and 80% (35/44) of the genes correctly predicted the inflammatory or non inflammatory phenotype of splenic DCs, confirming that the inflammatory signature selected in this study was also induced in DCs *in vivo* (Figure 6 and Figure S3). We were unable to confirm the induction of Il12b, Irf8 and Nfkb1 by LPS *in vivo*, possibly due to a lower expression of these genes *in vivo*.

## Discussion

The development and marketing of microarray platforms has led to extensive investigation of global gene expression profiles in health and disease. The expression profiling of diverse healthy tissues provides a comprehensive view of the range of transcriptional regulation in physiological conditions [43], [48]–[50]. Similarly, the identification of gene expression signatures indicative of disease subtypes improves our understanding of the molecular basis of disease [13], [14], [51]. Small sample size and the large number of measurements required for each sample currently limit the efficacy of gene expression profiling, leading to efforts to develop new analytical methods. Gene expression profiles have recently found applications in diagnosis, prognosis and the provision of predictive information, and in the classification of human cancers and inflammatory diseases [52], [53].

In this study, we used the random forest algorithm [54] to identify specific transcriptional signatures of inflammation in DCs and to evaluate whether these molecular signatures could be used to determine the activation state of DCs *in vitro* and *in vivo*. We found that the selected predictive genes upregulated during DCs activation fell into distinct functional classes, with major involvement in immune system processes (47%) cell differentiation (44%), metabolic process (42%) and cell death (31%). Gene Ontology analysis identified the genes involved in immune system processes as Psme2, Cd40, Ccl22, Il1b, Sqstm1, Tap1, Il6, Il12b, Ifit1, Ccr7, Irf8, Isg15, Nfkbia, Nfkb1, Stat5a and Cdkn1a. The genes involved in cell differentiation identified were Nfkbia, Skil, Traf1, Stat5a, Clic4, Gadd45b, Il12b, Irf8, Tnfrsf1b, Serpinb9, Sqstm1, Il6, Nfkb1, Cd40, Cdkn1a and Daxx. Gene Ontology analysis also identified genes involved in cell death, such as Traf1, Stat5a, Sqstm1, Il6, Nfkb1, Cd40, Cdkn1a, Gadd45b, Tnfrsf1b, Serpinb9 and Daxx. The downregulated predictive genes were identified as involved in cell division (Ccnb2, Kif20a, Cdc20), lipid metabolism (Daglb, Elovl6, Lta4h, Sgpp1), defense responses (hdac5, Il1rl1, Il18r1, Lta4h) and metabolic processes (Txndc16, Hdac5, Il1rl1, Daglb, Lta4h, Tep1, Sgpp1, H2-DMa, Eif4ebp2, Man2b1, Elovl6, Znrf2, Cdc20).

Most of the genes selected showed a sustained up-regulation or down-regulation suggesting that the processes they sustain, are important throughout the maturation process. Nevertheless, to deeply understand their functional role during DCs maturation, knock down studies should be performed on one or more genes of the signature.

Our findings clearly demonstrate that the gene expression profiling of DCs reliably distinguishes between activating and non activating stimuli. We validated our findings on an independent set of samples treated with molecules inducing the activity of different receptors and DC activation. Dexamethasone, Il10 and vitamin D were used as typical anti-inflammatory stimuli. They were generally classified as non activating signals. By contrast, TLR ligands and whole bacteria, which are widely recognized as inflammatory signals, were classified as such by this system. The signature expression profile identified here consists of 44 highly predictive genes. These 44 genes provide a unique gene expression profile indicative of activation in DCs and providing important biological insight into the host response mediated by DCs. Genes already identified as involved in inflammation and DC activation were selected, including those encoding the interleukins Il6, Il12b and Il1b [55], the chemokine Ccl22 [56], the membrane molecules Cd40, Cd83, Cd86, Icam1, and Ccr7 and the transcription factors Stat5a and Irf8, although the precise role of Irf8 in DC activation is currently unknown.

Some of the genes selected have no known role in inflammation. For example, the Pdzk1ip1 gene encodes a protein associated with various tumors when abundant. This protein seems to play a role in Akt activation and its gene was strongly induced by *L. monocytogenes* and LPS, whereas no overexpression was associated with poly I:C treatment. Thus, the induction of this gene is dependent on surface TLR stimulation. Rab20 encodes a protein that regulates intracellular trafficking and may play an important role in inflammation. Rab20 has been shown to interact with connexin 45 [43]. Il4i1 was recently identified as an oxidize active against *l*-amino acids with potential effects on lymphocyte proliferation. It is strongly induced by DC activation.

The genes encoding proteins involved in the NF-kB pathway were more strongly induced by bacteria than TLR ligands. Inhibitor genes, such as Nfkbia and Nfkbib, were also overexpressed after stimulation with bacteria, consistent with the activation of regulatory mechanisms that control the inflammation. The downregulated genes include a large proportion of membrane proteins, such as Il18r1, Il1rl1, Tspan8 and Tm7sf3, and genes encoding proteins with enzymatic activity, such as Man2b1 (an m-mannosidase), Lta4h (leukotriene A4 hydrolase), Txndc16 (thioredoxin domain-containing 16), Sgpp1 (sphingosine-1-phosphate phosphatase 1), Hdac5 (histone deacetylase 5). The role of this dowregulation is currently unknown and requires further investigation.

We used the dendritic cell line D1 to optimize the sensitivity and precision of our gene expression profiling. We decided to determine an inflammation signature for DCs rather than for any other type of leukocyte, because DCs link innate and adaptive immunity [57]. The prediction of DC activation state is therefore of potential value for the testing of exogenous molecules with potential anti-inflammatory or adjuvant activity in DCs, to favor the repression or induction of T-cell responses. We validated the inflammatory signature *in vivo*, by testing the response in splenic DCs from mice treated with LPS and dexamethasone. Most of the genes (80%) studied successfully characterized the activation state of splenic DCs, and differentiated the profile of these cells from that of DCs derived from mice treated with dexamethasone.

In conclusion, we used a meta-analysis of microarray data to identify gene modules predictive of DC activation. Accuracy and simplicity are essential characteristics of predictors for molecular assays. The predictive accuracy of predictors generally ranges from 65% to 100% (mean, 82%) [58]. It is therefore important to identify the best way to select a suitable classifier for data, to maximize accuracy. In this study we used random forest methods for the selection of genes and the classification of microarray data [54], [59]. The potential of array-based multidimensional predictors to outperform traditional parameters is increasing for biomarker discovery. The number of array-based studies is likely to increase exponentially, particularly in the field of inflammatory diseases. Such studies have been widely used for cancer classification [60], [61]. In this study, we identified and validated a prognostic gene expression signature in DCs associated with inflammation. The signaling events affected by many of the genes in this signature occur in pathways essential for the immune response and cell activation. These genes may therefore be suitable targets, alone or in combination, for trials of anti-inflammatory and adjuvant treatments. We have demonstrated that a genome-wide systems biology approach may have advantages over traditional methods for biomarker discovery. Moreover, the small number of genes in our signature makes it possible to use simple, conventional assays, such as quantitative reverse transcriptase-polymerase chain reaction [62]. The increasing availability of laboratory diagnosis by polymerase chain reaction has opened up new possibilities for genomic testing based on the use of genetic signatures, in routine clinical conditions.

## Materials and Methods

### Cell Culture

D1 cells [29] were maintained *in vitro* in Iscove's modified Dulbecco's medium (IMDM, Euroclone) supplemented with 10% heat-inactivated fetal bovine serum (Gibco, origin: Australia), 100 IU/ml penicillin, 100 µg/ml streptomycin, 2 mM L-glutamine (all from Euroclone) and 50 µM β-mercaptoethanol (Sigma) plus 30% R1 medium (supernatant from NIH3T3 fibroblasts transfected with GM-CSF).

MT2 cells are immortalized macrophages. They were derived from mouse thymus as previously described [47]. They were cultured in IMDM (Euroclone) supplemented with 5% heat-inactivated fetal bovine serum, 100 IU/ml penicillin, 100 µg/ml streptomycin, 2 mM L-glutamine (all from Euroclone) and 50 µM β-mercaptoethanol (Sigma).

### Microarray Dataset

We obtained published gene expression datasets for microarray experiments performed with D1 cells, dexamethasone (10^−8^ M), the live microorganisms *Schistosoma mansoni* (MOI 1∶200 parasites/cell) and *Leishmania mexicana* (MOI 1∶8) [23], [30], [31]. All the other samples were prepared specifically for this study. D1 cells were infected with *Listeria monocytogenes* EGD (MOI 1∶20; provided by P. Cossard, Pasteur Institute, France) or treated with various TLR agonists: rLPS (10 µg/ml, Alexis, serotype R515), CpG (10 µg/ml, Sigma), poly I:C (20 µg/ml, Amersham), Pam3Cys (1 µM, Sigma) and zymosan (500 ng/ml, Sigma). In summary, the data set is composed by: a) 14 untreated samples; b) 10 samples each of CpG, *Listeria monocytogenes*, Pam3cis and polyI:C at time points 2 h, 4 h, 8 h, 12 h and 24 h in duplicates; c) 8 samples each of *Leishmania amastigote*, *Leishmania promastigote*, *Shistosoma* SLA, *Shistosoma* EGGS and LPS at the time points 4 h, 8 h, 12 h and 24 h in duplicates; d) 6 samples of dexamethasone treated cells for the time points 4 h, 8 h and 24 h in duplicates; e) 15 zymosan treated samples at time points 2 h, 4 h, 8 h, 12, and 24 h in triplicates. The data set is composed in total of 115 microarrays.

### Microarray Assay

We harvested 10^7^ D1 cells in the immature state or after 4 h, 8 h, 12 h or 24 h of stimulation. Total RNA was isolated with Trizol Reagent (Invitrogen, Life Technologies, Karlruhe, Germany) and purified on a Qiagen RNeasy column (Qiagen, Hilden, Germany) to remove small fragments. RNA quality was assessed on an Agilent 2100 Bioanalyzer RNA 6000 Nano LabChip (Agilent Technologies, Palo Alto, CA). Only samples with intact total RNA profiles (retention of both ribosomal bands and the broad central peak of mRNA) were used for the microarray and quantitative RT-PCR gene expression analyses. *In vitro* transcription (IVT) products were generated and oligonucleotide array hybridization and scanning were carried out according to the instructions supplied by Affymetrix (Santa Clara, CA). We used 10 to 16 µg of total RNA from each sample and T7-linked oligo-dT primers for first-strand cDNA synthesis. The fragmented biotinylated cDNA (15 µg) was hybridized onto the MG-U74Av2 GeneChip (Affymetrix), using the recommended procedures for prehybridization, hybridization, washing and staining with streptavidin–phycoerythrin (SAPE).

### Microarray Data Analysis and Supervised Class Prediction

Array images were analyzed with the RMA algorithm [33]. Samples displaying a signal ratio >3.0 for the β-actin and GAPDH probe sets were considered to be poor-quality targets and were excluded from the dataset.

The final dataset contained the results for 115 arrays (79 DC samples subjected to pro-inflammatory stimuli and 36 samples with anti-inflammatory reagents). A single log scale normalized expression measure for each probe set was obtained from the low-level data files (CEL files), by the robust multiarray analysis (RMA) procedure [33]. The data were subjected to Z-score-based transformation. A diagnostic model was obtained by applying the random forest (RF) method to the training set [54]. The model was based on 1000 bootstrap samples of the training set, with 1000 classification trees generated with a view to classifying cases as “inflammatory” and “not-inflammatory” on the basis of microarray gene expression measurements. RF is a method of the “decision tree classifiers” family, but it works on a collection of trees (a ‘forest’) rather than a single tree. In a decision tree, each node represents an attribute — in our case, the probeset — and the terminal nodes (the ‘leaves’) represent the attribute producing the best separation between the classes –(“inflammatory” and “not inflammatory” in this analysis) of a dataset. RF feeds each tree with an independent subset of attributes from a training set and individual instances are classified by a voting procedure, with the majority of the decision trees in the collection indicating the appropriate classification. Finally, during the classification process, RF determines the relative importance of each attribute, through various methods, such as calculation of the Gini Index, which assesses the importance of the variable and carries out accurate variable selection.

### Quantitative Real-Time Polymerase Chain Reaction (qRT-PCR)

D1 cells were infected with *Listeria monocytogenes* EGD (multiplicities of infection, MOI, 1∶20) and *Lactobacillus paracasei* (MOI 1∶1000) or were treated with various stimuli: sLPS (10 µg/ml, Alexis, serotype SO55:B5), poly I:C (20 µg/ml, Amersham), zymosan (500 ng/ml, Sigma), dexamethasone (10^−8^ M, Sigma), vitamin D (10^−8^ M, Sigma), IL-10 (50 ng/ml, Immunok), IFNα (1000 U/ml, PBL Biomedical Laboratories) and Nimesulide (10 µM, Cayman).

We harvested 5×10^6^ D1 cells either at 0 h or after 24 h of stimulation. Total RNA was isolated with Trizol Reagent (Invitrogen) and purified on a Qiagen RNeasy column (Mini kit, Qiagen). DNase digestion was carried out in the column during RNA extraction (RNase-free DNase Set, Qiagen). RNA quantity and quality was evaluated spectrophotometrically (NanoDrop ND-1000 Spectrophotometer, Thermo Scientific). We reverse transcribed 1 µg of total RNA with random primers (High Capacity cDNA Reverse Transcription Kit, Applied Biosystems). Quantitative RT-PCR (qRT-PCR) was performed on 10 ng of total cDNA from independent samples, using primer sets specific for 54 selected genes and the 18s housekeeping gene. qRT-PCR was carried out on a 7500 machine (Applied Biosystems), with Power SYBR Green PCR Master Mix (Applied Biosystems). Assays were carried out in duplicate. Primers were designed with Primer3 software (http://frodo.wi.mit.edu/) and checked with other tools (BLAST, http://blast.ncbi.nlm.nih.gov/Blast.cgi; m-fold, http://mfold.bioinfo.rpi.edu/cgi-bin/dna-form1.cgi; IDT oligo analyzer,http://eu.idtdna.com/analyzer/Applications/OligoAnalyzer/). Primers were validated, and only primers with an amplification efficiency of 85 to 115% were accepted (Primm srl, Italy). Primer sequences are reported in Figure S4. The raw data (Ct, threshold cycle) were obtained with Applied Biosystems software. Relative mRNA levels were calculated by the 2^−ΔΔCt^ method (ΔCt  =  Ct_target_−Ct_18s_, ΔΔCt  =  ΔCt_stimulated_ − ΔCt_not treated_), using 18s as the housekeeping gene.

### *In Vivo* Experiment

Three mice per group of six-week-old C57BL/6 were injected with rLPS (50 µg/mouse, Alexis, serotype R515), dexamethasone (50 µg/mouse, Sigma), or with PBS as a control. The spleen was removed five hours later, and CD11c^+^ cells (DCs) were purified by magnetic bead separation (Miltenyi Biotec). C57BL/6 mice were purchased from Charles River and were maintained in our animal facility at the University of Milano-Bicocca. The *in vivo* experiment has been repeated two times. All experiments were performed using protocols approved by University of Milano-Bicocca Animal Care and Use Committee. Mice were housed in containment facilities of the animal facility and maintained on a regular 12∶12 hour light:dark cycle with food and water ad libitum.

## Supporting Information

Figure S1 Training and test sets, as used for the development of a classifier for the predictive analysis of microarrays. All samples were chosen based on the stimulus used for DC activation. The classifier, the random forest, was developed on the basis of two thirds of the samples (77 samples) and was then validated on the remaining one third (38 samples).
(0.36 MB TIF)

Figure S2 Selection of genes discriminating between DC phenotypes. A) We investigated the predictive value of genes by calculating the median level of expression for the gene in the inflamed and non inflamed samples (LPS, PolyI:C, zymosan, dexamethasone, IL-10 and vitamin D) and then calculating mean expression levels for that gene. B-C) These values were used to assess whether, for a particular stimulus (Listeria monocytogenes, Lactobacillus paracasei, nimesulide or IFNÎ±), the expression level of the gene concerned could be used to assign the sample to the correct class. A score of 1 was assigned if the expression level exceeded the mean value for inflammatory treatment or was below the mean level for anti-inflammatory treatment. A score of 0 was assigned in all other cases.
(10.47 MB TIF)

Figure S3 Selection of genes discriminating between different DC phenotypes in vitro and in vivo. Class predictor genes were identified on the basis of their mean levels of expression with known stimuli and their classification performance was determined in MT2 cells (A) and in ex vivo CD11c+ cells derived from the spleens of mice treated with LPS (B).
(1.16 MB TIF)

Figure S4 Primer sequences of house keeping and 54 genes used.
(0.03 MB XLS)
