# Correction: Gene Expression Profiles Identify Inflammatory Signatures in Dendritic Cells

**DOI:** 10.1371/annotation/53736770-ad30-4c6b-8279-d344a1232cc6

**Published:** 2010-06-15

**Authors:** Anna Torri, Ottavio Beretta, Anna Ranghetti, Francesca Granucci, Paola Ricciardi-Castagnoli, Maria Foti

The accession number for the gene discussed in this article was omitted. The accession number E-MEXP-2715 is registered with the ArrayExpress database and available at http://www.ebi.ac.uk/microarray-as/ae/browse.html?keywords=E-MEXP-2715.

