# Supplementary figures and images for: Gene Expression Profiles Identify Inflammatory Signatures in Dendritic Cells
Source: PLoS One. 2010 Feb 24;5(2):e9404. doi: 10.1371/journal.pone.0009404 (PMC2827557; doi:10.1371/journal.pone.0009404)

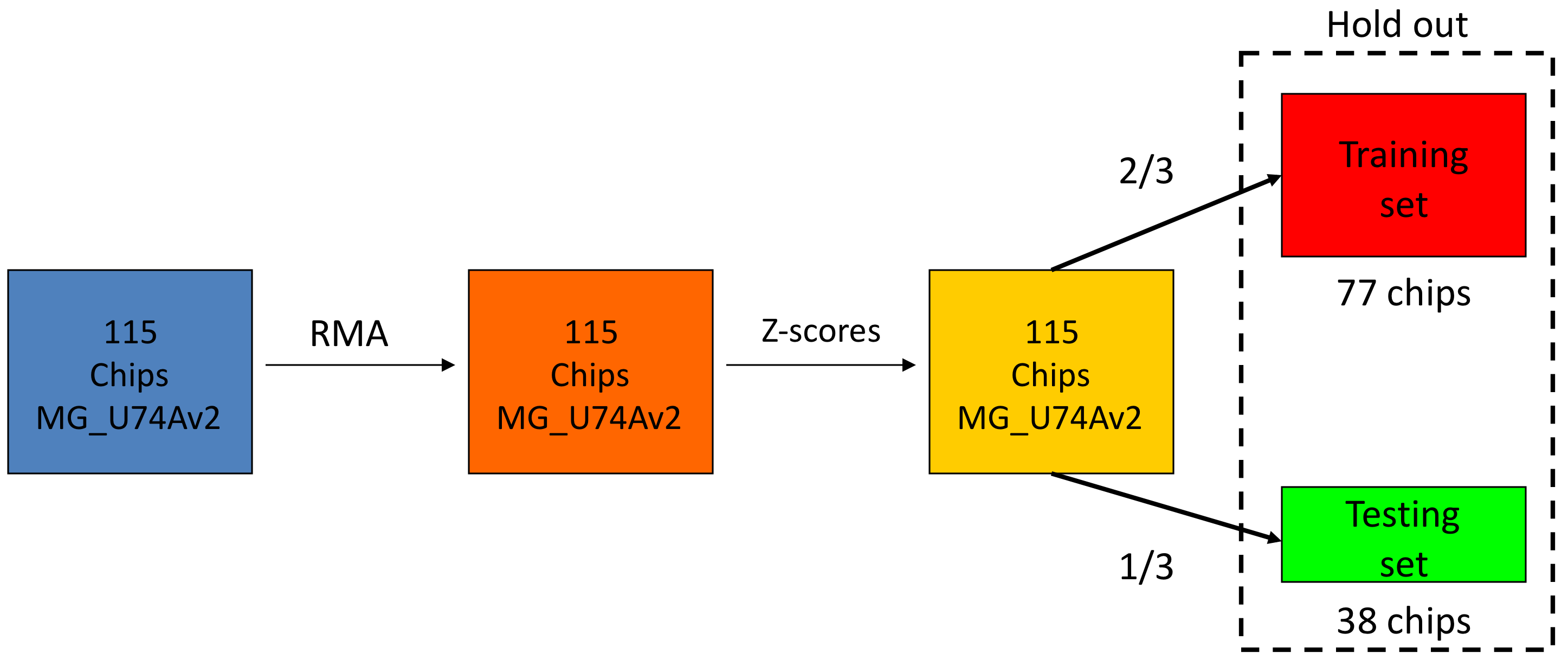

Supplement: Figure S1 — Training and test sets, as used for the development of a classifier for the predictive analysis of microarrays. All samples were chosen based on the stimulus used for DC activation. The classifier, the random forest, was developed on the basis of two thirds of the samples (77 samples) and was then validated on the remaining one third (38 samples). (0.36 MB TIF) [file pone.0009404.s001.tif]

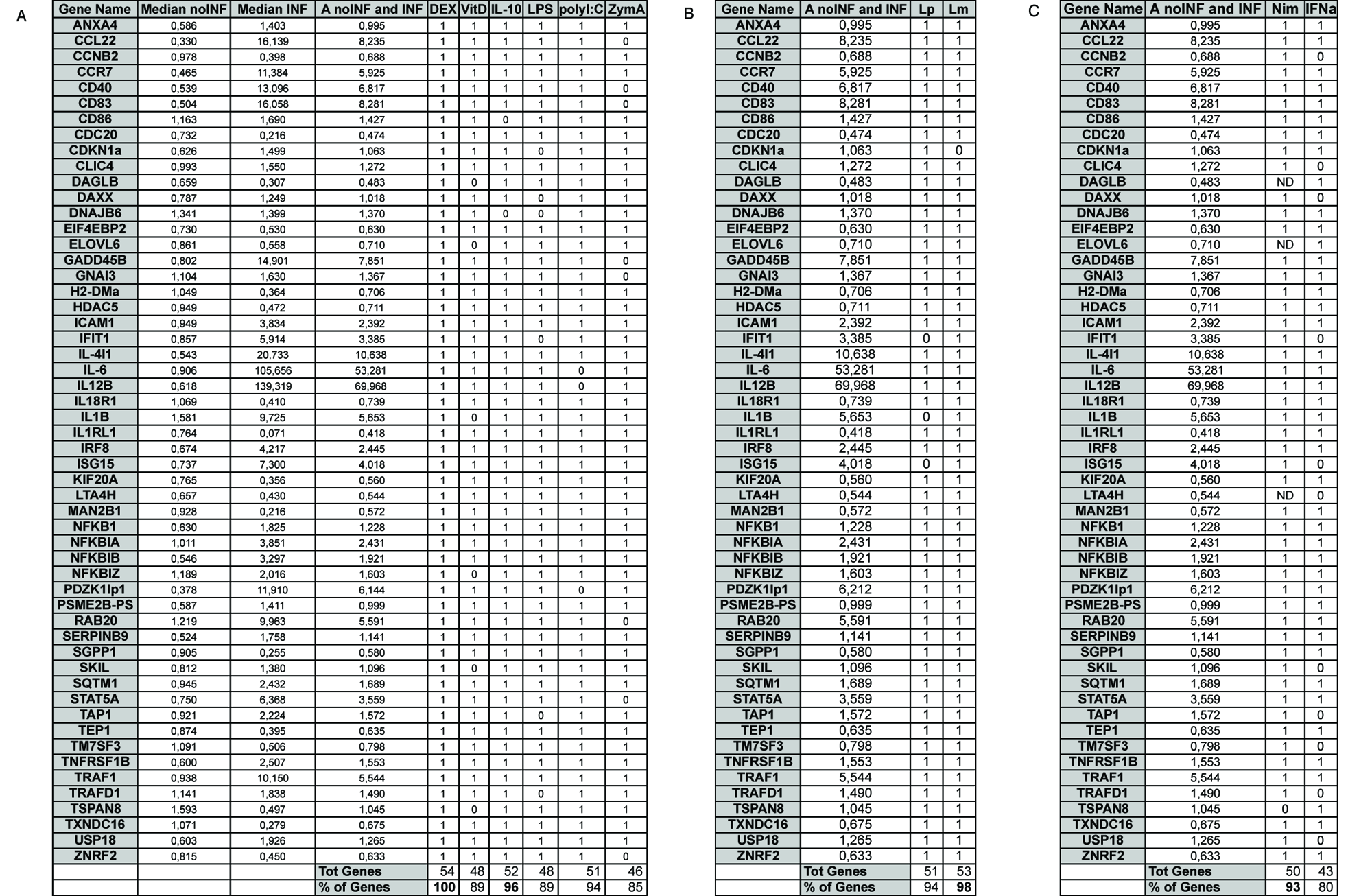

Supplement: Figure S2 — Selection of genes discriminating between DC phenotypes. A) We investigated the predictive value of genes by calculating the median level of expression for the gene in the inflamed and non inflamed samples (LPS, PolyI:C, zymosan, dexamethasone, IL-10 and vitamin D) and then calculating mean expression levels for that gene. B-C) These values were used to assess whether, for a particular stimulus (Listeria monocytogenes, Lactobacillus paracasei, nimesulide or IFNÎ±), the expression level of the gene concerned could be used to assign the sample to the correct class. A score of 1 was assigned if the expression level exceeded the mean value for inflammatory treatment or was below the mean level for anti-inflammatory treatment. A score of 0 was assigned in all other cases. (10.47 MB TIF) [file pone.0009404.s002.tif]

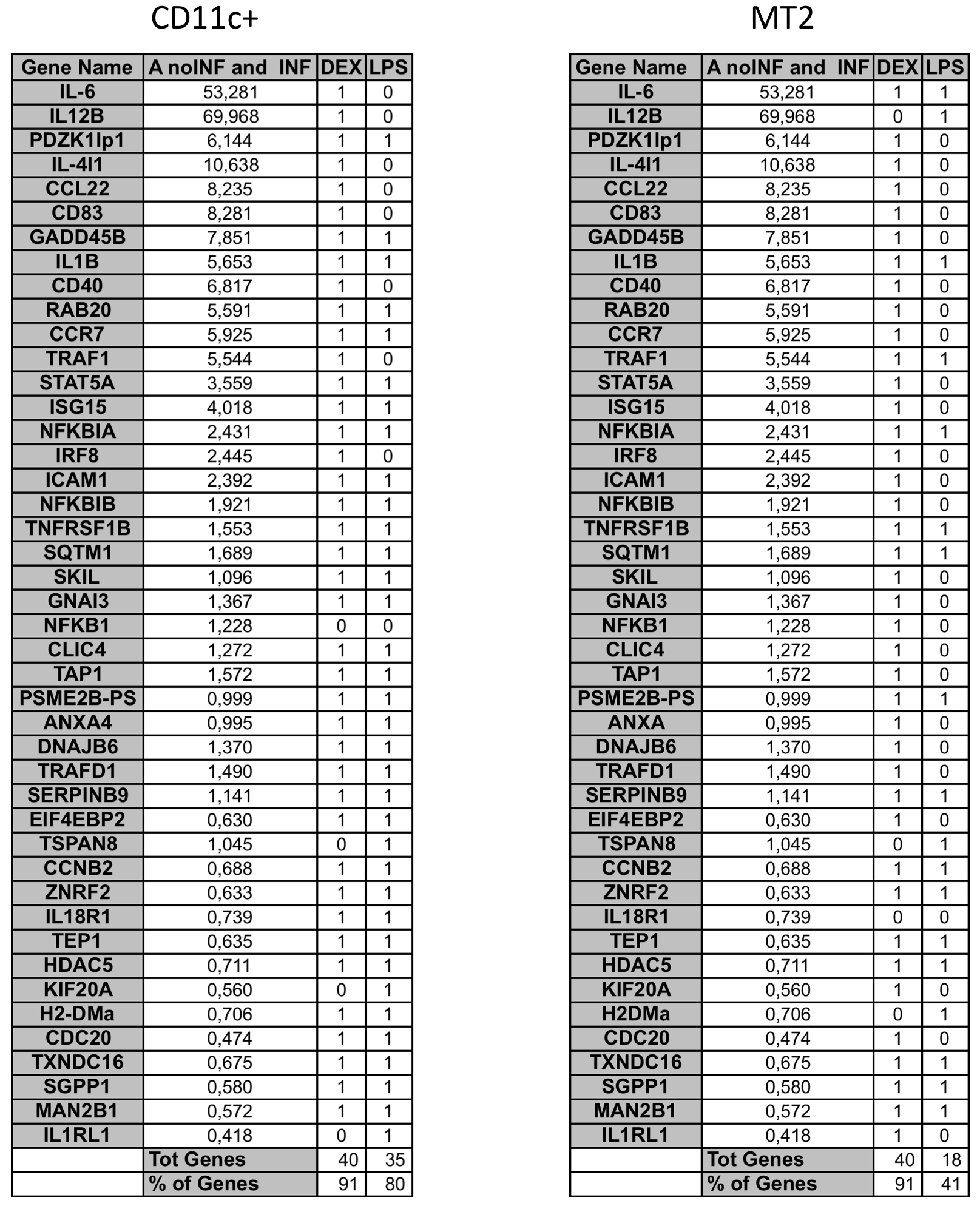

Supplement: Figure S3 — Selection of genes discriminating between different DC phenotypes in vitro and in vivo. Class predictor genes were identified on the basis of their mean levels of expression with known stimuli and their classification performance was determined in MT2 cells (A) and in ex vivo CD11c+ cells derived from the spleens of mice treated with LPS (B). (1.16 MB TIF) [file pone.0009404.s003.tif]
